# A case report of acute renal failure caused by anti-brucellosis treatment

**DOI:** 10.1097/MD.0000000000037560

**Published:** 2024-03-29

**Authors:** Yuanyi Miao, Xuegang Li

**Affiliations:** a Department of Nephrology, Weihai Municipal Hospital, Weihai, China

**Keywords:** Acute renal failure, brucellosis, contrast, gentamicin, rifampicin

## Abstract

**Rationale:**

Rifampicin, as a main chemotherapy drug treating brucellosis, is widely used in clinical practice. Rifampicin-associated ARF is not rare, especially in those rifampicin re-exposure patients. However, this was rare complication of severe renal involvement due to multiple factors including rifampicin, nephrotoxic gentamicin, and contrast medium, and few studies have reported it.

**Patient concerns:**

A 59-year-old male presented to our hospital with acute renal failure (ARF) caused by anti-brucellosis treatment with rifampicin (675 mg/day), gentamicin (320 mg/day), and doxycycline (200 mg/day). He had a contrast-enhanced CT of the upper abdomen before the onset of. After stopping rifampicin and undergoing integrated therapy, the patient’s renal function gradually recovered.

**Diagnoses:**

Considering that the patient had a history of using rifampicin for pulmonary tuberculosis in the past, based on the examination results, the patient was diagnosed with rifampicin-associated ARF.

**Interventions:**

Symptomatic treatment such as hemodialysis, and anti-brucella treatment with doxycycline and moxifloxacin were given.

**Outcomes:**

The patient had significant anuric and polyuric periods and acute tubular necrosis is considered. After treatment, his renal function and urine volume returned to normal, and *Brucella melitensis* was not isolated from blood cultures.

**Lessons:**

The case reveals that severe renal involvement due to multiple factors including rifampicin, nephrotoxic gentamicin, and contrast medium. Misdiagnosis and mistreatment can deteriorate the patient’s condition. Renal function should be closely monitored in the susceptible patients. Early recognition can provide appropriate therapy to patients. If unexplained renal failure during the use of rifampicin, especially in those rifampicin re-exposure patients, rifampicin-associated ARF should be considered.

## 1. Introduction

Brucellosis is an infectious zoonotic disease circulating most frequently by ingestion of animal products, such as unpasteurized dairy products, or exposure to the tissue or body fluid. Brucellosis is disease with multiorgan involvement and there are many clinical studies about it.^[1–8]^ Many patients are misdiagnosed with arthritis or other nonspecific infections. The disease is the most common zoonotic disease in the world and an important public health problem in many settings.^[9,10]^

The goals of treatment for brucellosis are disease control and prevention of complications, recurrence, sequelae, and death.^[11,12]^ For adults without spondylitis, neurobrucellosis or endocarditis, the preferred treatment regimen is doxycycline plus aminoglycoside.^[13,14]^ However, many prefer doxycycline-rifampicin for reasons including oral administration is more convenient than parenteral administration, lower cost, tolerability may be better than aminoglycosides which are nephrotoxic and ototoxic.^[13,15]^ For adults and pediatric patients over 8 years old with spondylitis, we generally administer streptomycin on the first 14–21 days or gentamicin, doxycycline plus rifampicin on the first 7–14 days for at least 12 weeks.^[16–19]^ Patients with renal failure are treated with doxycycline plus rifampicin.^[20]^

Rifampicin has a broad antibacterial spectrum and a powerful effect. The antibacterial mechanism is that it inhibits bacterial DNA-dependent RNA polymerase.^[21]^ Common adverse reactions include gastrointestinal effects, central nervous system effects, dermatologic effects, hematologic effects, and pulmonary toxicity.^[22]^ In 1971, Poole et al first reported that rifampicin could cause acute renal failure (ARF).^[23]^ Subsequently, reports of adverse reactions such as liver and kidney dysfunction, gastrointestinal reactions, blood system damage, and drug-induced rashes caused by rifampicin gradually increased. It is clinically speculated that the possible mechanism of ARF is related to the production of rifampicin antibodies. At present, it is believed that rifampicin may be mainly caused by the immune mechanism, which is different from the general drug-induced renal damage mechanism.

## 2. Case presentation

The patient developed left knee joint pain without obvious incentive more than 5 years ago. He did not systematically check and treat it. One year ago, he went to the orthopedic surgery department of Weihaiwei People’s Hospital and underwent left knee joint replacement surgery. The operation was successful. Recently, he felt purulent secretions at the joint replacement surgery site. He went to the hospital again and underwent left knee arthroscopy. The diagnosis was sinus formation after left knee replacement surgery and the joint secretions were collected. The culture examination was “Brucella,” and the renal function was normal at that time.

After being discharged from the hospital, for further treatment, he was admitted to the Weihai Chest Hospital on June 23, 2022, and was given triple anti-brucella treatment with doxycycline (200 mg/day), rifampicin (675 mg/day), and gentamicin (320 mg/day). During hospitalization, he had a contrast-enhanced CT of the upper abdomen showed: multiple low-density lesions in the liver, considering liver cyst; splenomegaly; and left kidney cyst. CT of chest, lumbar spine, and hip joint showed: bilateral lung lesions, considering tuberculosis; focal emphysema and bullae in both lungs; bilateral pleural thickening; lumbar vertebral bone changes, considering brucellosis; lumbar degenerative changes; and bilateral hip joint degeneration. After 1 week of gentamicin application discontinued, rifampicin was discontinued after 1 week of adverse drug reactions. Anuria, nausea, and vomiting occurred 5 days before admission, vomited 3 times, the vomit was all stomach contents, no hematemesis, no chills, no fever, no dizziness, no headache, no cough, no expectoration, no chest tightness, no suffocation, no palpitation, no shortness of breath. Liver and kidney function check on July 4, 2022: albumin 33.1 g/L, aspartate aminotransferase 46 U/L, urea nitrogen 33.01 mmol/L, calcium 1.88 mmol/L, carbon dioxide 17.4 mmol/L, creatinine 700 µmol/L. Myocardial enzymes: creatine kinase 559 U/L, lactate dehydrogenase 648 U/L, α-hydroxybutyrate dehydrogenase 571 U/L. Blood routine: lymphocyte count 0.46 × 10^9^/L, neutrophil ratio 79.0%, hemoglobin 93 g/L, platelets 103 × 10^9^/L, C-reactive protein (CRP) 20.81 mg/L. Procalcitonin 72.85 ng/mL, erythrocyte sedimentation rate (ESR) 23 mm/h. He was considered ARF after consultation with our department. The discharge diagnosis: ARF; brucellosis; brucellosis spondylitis; lumbar spine degeneration; bilateral hip joint degeneration; emphysema; bullae; old tuberculosis; intrahepatic nodules; liver cyst; and left kidney cyst. After the patient was discharged from the hospital for further treatment, he was admitted to our department with ARF. Since the onset of the disease, the patient has been in good spirits, with reduced diet, poor sleep, normal stool, and no significant change in weight.

The patient reported that he had been previously worked as a shepherd. Physical examination revealed a man with pale skin with a temperature of 36.1°C and a pulse rate of 72/minute. His spleen was 1 cm below the costal margins, respectively. The left knee joint can be seen with the dressing in place, and a nodule with a length of about 3 × 3 cm can be seen on the radial side of the left elbow joint. He has a history of pulmonary tuberculosis and improved after taking regular medication (including rifampicin). He denied the history of hepatitis, malaria, cardiovascular disease, diabetes, cerebrovascular disease, mental illness, trauma, blood transfusion, and drug allergies.

Examinations check on July 5, 2022: direct bilirubin 10.3 µmol/L, total protein 60.9 g/L, albumin 34.5 g/L, aspartate aminotransferase 43 U/L, glutamyl transpeptidase 94 U/L, adenosine deaminase 25.8 U/L, urea nitrogen 34.01 mmol/L, creatinine 800 µmol/L, uric acid 684 µmol/L, sodium 134 mmol/L, potassium 3.80 mmol/L, calcium 1.87 mmol/L, carbon dioxide 16.9 mmol/L, creatine kinase 469 U/L, lactate dehydrogenase 575 U/L, α-hydroxybutyrate dehydrogenase 500 U/L; Prothrombin time 13.1 seconds, PT activity 82%, international normalized ratio 1.09, activated partial thromboplastin time 30.1 seconds, thrombin time 14.5 seconds, fibrinogen 3.41 g/L, D-dimer 1.84 mg/L, procalcitonin 16.75 ng/mL, hepatitis B surface antigen 0.01 IU/mL, syphilis antibody 0.19 s/co, AIDS antibody 0.01 s/co, Hepatitis C antibody 0.1 s/co. Because of his oliguria and progressive increase in creatinine, he was carried on right internal jugular venous puncture and catheterization and started hemodialysis.

Consulting with the orthopedic department, he was recommended to reduce walking, go down with crutches, review the anterior and lateral X-rays of the left knee joint, and consult an infectious disease department to assist in diagnosis and treatment. Consulting with the infectious disease department, he was recommended to use drugs with low nephrotoxicity (such as doxycycline, ceftriaxone, moxifloxacin, etc.). Basis on comprehensive consideration, we gave him anti-brucella treatment with doxycycline (200 mg/day) and moxifloxacin (400 mg/day).

Examinations check on July 6, 2022: carbon dioxide binding capacity 24.6 mmol/L, urea nitrogen 30 mmol/L, total calcium 1.97 mmol/L, phosphorus 1.26 mmol/L, creatinine 785.1 µmol/L, uric acid 631.6 µmol/L, total protein 58.5 g/L, albumin 30.7 g/L, alanine aminotransferase 22 U/L, aspartate aminotransferase 27.2 U/L, creatine kinase 278.3 U/L, lactate dehydrogenase 440.5 U/L, α-hydroxybutyrate dehydrogenase 400 U/L. Blood routine: white blood cell count 4.48 × 10^9^/L, hemoglobin 92 g/L, platelet count 95 × 10^9^/L, ESR 21 mm/h.

Examinations check on July 7, 2022: urine routine examination plus sediment: urine protein 1+, red blood cell 33.5/µL, complement C3 0.93 g/L, complement C4 0.29 g/L. Direct antiglobulin test was negative. Blood routine: white blood cell count 4.57 × 10^9/^L, hemoglobin 90 g/L, platelet count 111 × 10^9^/L. Color Doppler: left kidney size 139 mm × 70 mm, right kidney size 128 mm × 74 mm, slightly larger in size, plump in shape, still smooth membrane, enhanced cortical echo, and unclear boundary with medulla. Color Doppler flow imaging showed that the blood supply was acceptable. Both kidneys were large, diffuse lesions, enlarged prostate with calcification, and no obvious abnormality in the ureter. Echocardiography: mild enlargement of the left atrium, mild dilation of the aortic sinus and ascending aorta, aortic valve regurgitation (small amount), decreased left ventricular diastolic function.

Examinations check on July 8, 2022: blood routine: white blood cell count 4.98 × 10^9^/L, hemoglobin 92 g/L, platelet count 175 × 10^9^/L. Urea nitrogen 16.6 mmol/L, total calcium 1.99 mmol/L, creatinine 591.8 µmol/L, total protein 59.1 g/L, albumin 33.5 g/L, alanine aminotransferase 15.7 U/L, aspartate aminotransferase 17.7 U/L, creatine kinase 120.1 U/L, lactate dehydrogenase 362.9 U/L, α-hydroxybutyrate dehydrogenase 360 U/L. Perinuclear antineutrophil cytoplasmic antibody IgG negative, cytoplasmic antineutrophil cytoplasmic antibody IgG negative, anti-proteinase 3 antibody 1CU, anti-myeloperoxidase antibody 1CU, anti-glomerular basement membrane antibody 1.27, antinuclear antibody IgG negative, anti-double-stranded DNA test negative, anti-double-stranded DNA antibody IgG 8.2I U/mL.

Rechecked on July 9, 2022: CRP 14.26 mg/L, ESR 20 mm/h, Procalcitonin 1.97 ng/mL. Examinations check on July 12, 2022: Blood routine: white blood cell count 5.4 × 10^9^/L, hemoglobin 87 g/L, platelet count 236 × 10^9^/L. Urea nitrogen 20.38 mmol/L, creatinine 809.1 µmol/L, uric acid 476.7 µmol/L, total protein 65.4 g/L, albumin 36.3 g/L, alanine aminotransferase 18.8 U/L, aspartate aminotransferase 15.7 U/L, lactate dehydrogenase 329.5 U/L, α-hydroxybutyrate dehydrogenase 281 U/L. Fecal occult blood negative.

Examinations check on July 14, 2022: urea nitrogen 18.27 mmol/L. Creatinine 639.9 µmol/L. Examinations check on July 15, 2022: urea nitrogen 13.84 mmol/L, creatinine 363.4 µmol/L. Examinations check on July 17, 2022: urea nitrogen 19.9 mmol/L, creatinine 383.8 µmol/L, folic acid 23.47 nmol/L, vitamin B12 33.6 pg/mL, ferritin 249.5 ng/mL, procalcitonin 0.26 ng/mL. Blood routine: white blood cell count 3.52 × 10^9^/L, hemoglobin 96 g/L, platelet count 154 × 10^9^/L. CRP 3.19 mg/L, ESR 21 mm/h. Brucella test: Negative for Brucella.

**Figure 1.**
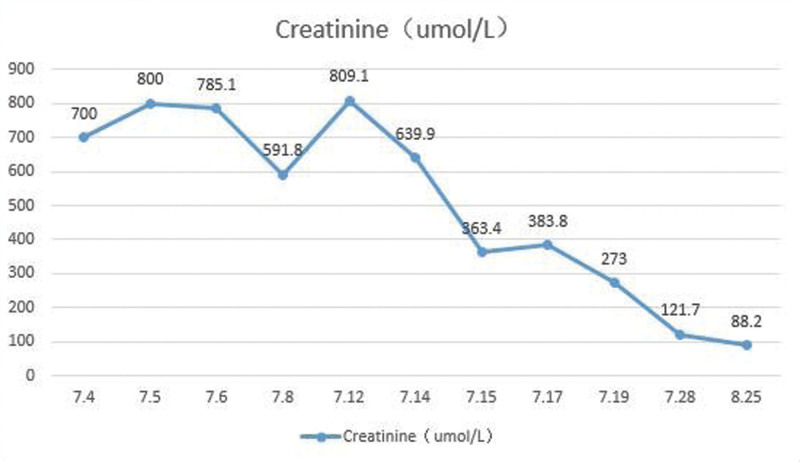
Changes in creatinine. After treatment, the patient’s renal function gradually recovered.

Examinations check on July 19, 2022: urea nitrogen 18.14 mmol/L, total calcium 2.21 mmol/L, phosphorus 1.4 mmol/L, creatinine 273 µmol/L, total protein 76 g/L, albumin 43.3 g/L, alanine aminotransferase 15.5 U/L, aspartate aminotransferase 14.6 U/L, alkaline phosphatase 74.7 U/L, lactate dehydrogenase 214.7 U/L. After treatment, the patient’s renal function (Fig. 1), myocardial enzymes and CRP gradually recovered, and the internal jugular vein catheter was removed and discharged.

Rechecked on July 28, 2022: white blood cell count 3.94 × 10^9^/L, hemoglobin 103 g/L, platelet count 177 × 10^9^/L, CRP 5.54 mg/L, urea nitrogen 9.1 mmol/L, total calcium 2.26 mmol/L, creatinine 121.7 µmol/L. Brucella test was negative. Urine routine examination plus sediment: urine protein−, red blood cell 29.8/µL. Rechecked on August 25, 2022: creatinine 88.2 µmol/L, urea nitrogen 5.8 mmol/L. Urine routine examination plus sediment: urine protein−, red blood cell 1.9/µL

## 3. Discussion

Brucellosis can involve in any organ system.^[2,7,8,24,25]^ Osteoarthropathy is the most common form of focal brucellosis, accounting for up to 70% of patients with brucellosis.^[4,26,27]^ Osteoarthropathy include peripheral arthritis, sacroiliitis, and spondylitis. The most commonly affected sites are the sacroiliac joints and the spinal joints.^[28]^ Peripheral arthritis and sacroiliitis appear in acute cases. Peripheral arthritis usually involves the knee, hip, and ankle joints.^[26,27,29]^ Brucellosis can be diagnosed by finding Brucella in culture (blood, body fluid or tissue).^[30]^ The patient had spinal, sacroiliac, and knee joint involvement and Brucella was cultured in his joint effusion.

General principles for the treatment of brucellosis include antibiotics active in the acidic intracellular milieu, such as doxycycline and rifampicin. Due to high recurrence rates with monotherapy, combination therapy can shorten treatment periods.

Rifampicin, a small molecule compound (molecular weight <1000 Daltons) is antigenic only when bound to plasma proteins. In the process of rifampicin treatment, as a hapten, rifampicin can bind proteins or attach to cell membranes to stimulate the body to produce antibodies, mainly IgG and IgM. Rifampicin binds to antibodies in vivo to form antigen–antibody complexes, and further binds to major histocompatibility complex (MHC) class I antigens on the cell surface, leading to cell damage with the participation of complement. Tubular epithelial cells express the same MHCI antigens while glomerular epithelial cells express less MHCI antigens, which may be the reason for acute tubular necrosis (ATN) and normal glomeruli. Rifampicin-induced renal injury can be manifested in five pathological types: ATN; acute interstitial nephritis (AIN); crescentic glomerulonephritis; light chain proteinuria; and section segmental necrotizing glomerulonephritis. Among them, ATN and AIN are the most common.^[31]^

**Figure 2.**
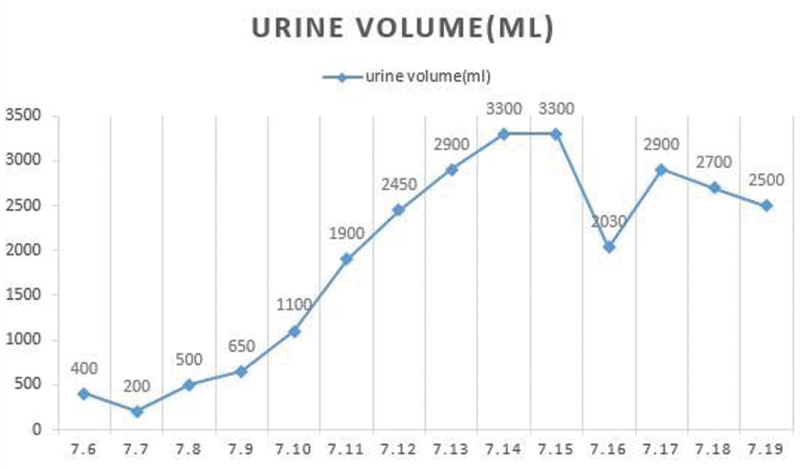
Changes in urine volume. The patient has significant anuric and polyuric periods.

The patient’s decreased hemoglobin, thrombocytopenia, and ARF are unknown at present, it needs to be differentiated from ATN, AIN, and contrast-induced acute kidney injury (CI-AKI). ATN: patients often use gentamicin and other nephrotoxicity drugs can cause ARF, manifested as oliguria, anuria, renal tubular dysfunction, and low specific gravity urine. AIN: It can be caused by drugs, manifested as rash, fever, eosinophilia, and eosinophils can be found in urine. An imaging examination shows that both kidneys are normal or enlarged. TMA: Hemolysis, thrombocytopenia, and organ involvement caused by microthrombosis. The patient’s renal failure progresses rapidly. CI-AKI: The main clinical manifestations of CI-AKI include early mild elevation of serum creatinine. Serum creatinine elevation is generally observed within 24–48 hours after exposure to iodinated contrast agent, and the elevation is usually mild. Serum creatinine typically begins to decline toward baseline within 3 to 7 days after contrast exposure. Since AKI is usually mild, most patients have no oliguric manifestations.^[32,33]^ He has significant anuric and polyuric periods (Fig. 2). ATN is considered.

The limitation of this case is that the patient’s condition gradually improved, and no renal biopsy was performed to confirm the pathological diagnosis. Clinical reports on multiple factors-associated ARF are relatively rare, and large-scale, multicenter clinical studies are still needed to further verify whether nephrotoxic gentamicin and contrast medium can aggravate renal failure. We present this case to emphasize rifampicin-associated ARF is not rare, especially in those rifampicin re-exposure patients. However, this was rare complication of severe renal involvement due to multiple factors including rifampicin, nephrotoxic gentamicin, and contrast medium. Renal function should be closely monitored in the susceptible patients.

## Acknowledgments

Thanks for the help of colleague Haina Yu. Thank the patient for giving consent to participate and consent for publication.

## Author contributions

**Data curation:** Yuanyi Miao.

**Formal analysis:** Yuanyi Miao.

**Writing—original draft:** Yuanyi Miao.

**Writing—review & editing:** Xuegang Li.
